# Novel Designed Surgical Drapes Reducing Fluid Permeability in the Surgical Critical Area of a Sterile Operation Interface: A Randomized Controlled Trial

**DOI:** 10.1155/2023/9295307

**Published:** 2023-03-16

**Authors:** Chang-qing Liu, Hong-fei Ren, Chen Wang, Ji Li, Li Tang, Jing-jing An, Ka Li, Yan-li Luo

**Affiliations:** ^1^Department of Operating Room of West China Hospital, West China School of Nursing, Sichuan University, Chengdu, Sichuan, China; ^2^Nursing Key Laboratory of Sichuan Province, Chengdu, Sichuan, China; ^3^Department of Gastroenterology of West China Hospital, Sichuan University, Chengdu, Sichuan, China; ^4^West China School of Nursing, Sichuan University, Chengdu, Sichuan, China

## Abstract

**Aim:**

To compare the impact and cost effects of medical long fiber polyester drapes and cotton fabric drapes on operative sterile operation interfaces.

**Background:**

The comparison of the properties of the commonly used surgical drapes materials in terms of leakage, device slip, and prevention of intraoperative adverse events is not clear.

**Method:**

A prospective randomized controlled study was conducted in the operating room of a tertiary hospital in Chengdu, China. A total of 400 patients who underwent urology surgery were enrolled and randomly divided into two groups by computer, the study group (200 cases) selected the new long-fiber polyester cloth, while the control group (200 cases) selected conventional cotton fabric surgical drapes during the operation to maintain a sterile operating interface. The impermeability and water absorption of surgical drapes, the rate of device slip and skin scald in surgical patients, and the cost effect of the two kinds of surgical drapes were compared.

**Results:**

The long fiber polyester surgical drapes were superior to conventional cotton cloth in water absorption (g/m^2^) (835 ± 15.8 VS 225 ± 21.0, *t* = 328.261, *P* < 0.001), preventing surgical site infections (2.5% VS 8.0%, *χ*^2^ = 6.081, *P*=0.014), device slip (7.5% VS 17.0%, *χ*^2^ = 8.396, *P*=0.004), patients from burning (0 VS 1, Fisher *P*=1.0), and total cost per use ($) (0.83 VS 0.96–1.09).

**Conclusion:**

Long fiber polyester fabric has a stronger antipenetration ability of fluid and microorganisms thus forming an effective protective barrier. It also has strong hygroscopicity, and its special design can prevent the occurrence of sliding of surface instruments and skin scald in patients. In addition, its cost effect is superior. *Implications for Nursing Management.* Operating room nursing managers can introduce long fiber polyester drapes into the selection of medical textiles to construct aseptic surgical barriers and prevent surgical site infection.

## 1. Introduction

Surgical site infection (SSI) is one of the most common complications after surgery and a major factor (≥5%) in patients' remorbidity and readmission [1]. It has been reported that approximately 300,000 to 500,000 SSI cases occur annually in the United States, of which 10% to 16% occur after clean-to-decontamination surgery and 2% after discharge [2]. SSI in Europe is approximately 1.5%∼20% [3], and approximately 60,000 to 128,000 cases occur annually in Germany alone [4]. SSI in patients undergoing surgery will prolong their hospital stay, increase their medical costs, and even increase their mortality [5]. A study has shown that over 75% of patients dying after surgery are directly related to SSI [6]. Another study pointed out that SSI ranked first in the incidence of nosocomial infections in surgical patients (38%) [7]. Therefore, SSI control and prevention are important indicators of operating room quality management.

The operating room is a department for the rescue of all kinds of critical patients and the operation of the whole hospital as well as for the control and prevention of nosocomial infection [8]. Drapes have been used during invasive procedures to maintain the sterility of environmental surfaces, equipment, and patients [9]. A surgical drape is a necessary and frequently used sterile surgical appliance in the operating room, which is mainly used to establish the barrier of the surgical sterile area [10]. Surgical drapes play a pivotal two-way protective role in the barrier system against wound infection, not only reducing the risk of contact with pathogenic microorganisms of medical staff but also blocking the spread of various microorganisms on the clothing and skin of medical staff to the surgical incision and protecting the surgical field from environmental pollution. It is the duty of healthcare workers to provide an infection-free environment for patients undergoing surgery. In the perioperative setting, prevention of contamination for both surgical patients and personnel is a prime objective. The assessment and improvement of materials with appropriate barrier efficacy for consumption in the operating room is still under investigation [11].

The potential of surgical drapes to prevent contamination mainly depends on the physical properties. For instance, the penetration of microorganisms will be smaller through several layers of the same fabric compared to one layer [12]. In addition, using single-layer surgical drapes not only increases the number of laundry and sterilization cycles but also increases bacterial penetration. Hence, single-layer drapes due to the lower barrier index and high cumulative penetration ratio are not suggested for a long time and risky operation. A study's result [13] demonstrated that three-layer materials of surgical drapes have higher protective performance such that even after repeated laundry and sterilization processes, they present a higher barrier index. There are several factors that can affect the protective effect of surgical drapes, including dampness, the number of washings given, the kept environment, the sterilizations given, and the span of surgical operations in which it is used [14]. Researchers found that there should be some specific characteristics in surgical gowns and drapes, such as the capacity to resist scratches, tears, flames, liquid strikes, bacterial strikes, and any kind of discharge [15].

Traditional surgical drapes are mostly made of cotton cloth, which has a relatively short period of validity after sterilization, poor impermeability, and easy infiltration by washing fluid, blood, urine, and sweat [16]. In addition, with the extension of time, cotton threads or loose floc may fall into the wound and even carry bacteria to cause incision contamination. Although disinfection and sterilization technology is currently greatly improved, a study showed that cotton is woven surgical drapes often have a high wear rate and significantly reduced barrier after washing 25 times, resulting in increased surgical infection risk for patients [17]. One of the concerns in applying traditional cotton surgical drapes is the reduction of their resistance to bacterial penetration, especially in the wet state, after repeated laundering and sterilizing processes [18]. In developed countries, long-fiber polyester cloth has been widely used in clinical practice and has basically replaced cotton-woven surgical drapes in recent years. At present, the raw materials of surgical drapes used are generally cotton cloth in China. The main disadvantage is poor moisture resistance, ease of soaking in blood and water, inability to block bacteria, and self-protection once soaked [19]. A study has illustrated that the positive rate of incision bacterial culture in the surgical group using polyester filament fiber drapes was only 15.6%, which was significantly lower than 45.5% in the cotton group (*P* < 0.05). The infection rate of postoperative incision in the control group of cotton was 12.1%, which slightly higher than that in the polyester filament fiber group (9.4%) [20]. Other study's results implied that compared with traditional cloth surgical drape (*n* = 218), nonwoven surgical drape (*n* = 212) can significantly reduce the bacterial infection of the incision, the rate of dressing wetting in emergency cesarean, and reduce maternal heat loss (*P* < 0.05) [21]. The antibacterial properties of long fiber polyester cloth and traditional cotton cloth surgical drapes were discussed in the relevant studies at home and abroad, which proved the feasibility of their safe use. However, the factors such as antifluid penetration, preventing the instrument from slipping, and preventing the patient from burning have not been researched.

In recent years, with the continuous increase in the number of operations in Chinese hospitals, the use of cotton fabric drapes and cleaning and packaging are facing an increasing number of challenges, which have also become a large part of the hospital operating costs. At the same time, with the increasing incidence of infectious diseases such as HIV, hepatitis B, and hepatitis C, the occupational exposure risk of medical staff has also increased year by year [22]. The choice of nonwoven fabric as nursing materials by many medical institutions is becoming a development trend. However, the use of disposable non-woven fabric will cause more medical waste and increase the cost related to surgery. A systematic review [23] suggests that there is currently no evidence to support a distinction between reusable or disposable surgical towels to reduce the risk of SSI in orthopedic and spinal surgery. At present, cotton fabric and disposable nonwoven fabric are widely used for domestic medical packaging and surgical towel materials. However, they may have some drawbacks, such as poor permeability resistance, high production of medical waste, and high disinfection and disposal costs. Therefore, finding a high-quality reusable new material with relevant surgical drapes standards will become an urgent need for the maintenance of aseptic surgical interfaces.

In this study, we introduce a new surgical towel material, long-fiber polyester surgical drapes, which was intended to be used in the surgical patients of a urology surgery of a tertiary hospital (large doses of surgical rinsing fluid, high waterproof requirements of surgical drapes, etc.), to compare the advantages and disadvantages and the cost effect of cotton woven cloth and long-fiber polyester cloth, such as liquid leakage resistance, water absorption, instrument slip prevention, skin burn prevention, and other properties during surgery, and the influence on the postoperative infection rate of patients, so as to provide a reference for rationally selecting surgical drapes, ensuring the safety of surgical patients, reducing SSI, and reducing surgery-related costs.

## 2. Methods

### 2.1. Study Design

We conducted a prospective, randomized, and controlled trial to analyze and compare the impact and cost effects of medical long-fiber polyester cloth and cotton fabric cloth on operative sterile operation interfaces in the operating room.

### 2.2. Sample and Grouping

The study was conducted in the operating room of a tertiary hospital in Chengdu, China. The sample size was calculated by comparing the independent sample rates of the two groups, and the postoperative surgical site infection (SSI) rate was used as the main index to calculate the sample size. A study reported that the postoperative SSI rate of patients with long-fiber polyester surgical drapes was 8.82%, while that of patients with cotton surgical drapes was 20.59% [24]. The calculated sample size was each 139 cases in the two groups. Considering the 20% loss to followup rate, the sample size of the study group and the control group was 167 cases, respectively. In view of the feasibility of statistical analysis and sample inclusion, the sample size of the study group and control group was planned to be 200 cases, respectively, for a total of 400 cases. Finally, a total of 400 patients undergoing urology surgery were included as the research subjects from July to October 2022 and were randomly divided into two groups with 200 cases in the study group and 200 cases in the control group by computer. In the study group, the surgical patients used long fiber polyester fabric surgical drapes, while the patients used conventional cotton surgical drapes in the control group. Random numbers were generated by the computer to implement allocation hiding. The serial numbers of the patients to be included in the study were then assigned by a computer. We interviewed patients and their primary caregivers or families and then explained the study protocols on the day before surgery, and the patients were successively enrolled in the study by researchers according to the serial numbers and the order of inclusion. Data analysts were blinded in this study.

The criteria for inclusion and exclusion of subjects were as follows: (1) inclusion criteria: ① the patients undergoing elective surgery under general anesthesia in the urological operating room; ② the patients whose preoperative skin preparation meeting the requirements of surgical disinfection standards and expected to achieve a clean incision standard were evaluated by the surgeon and included; ③ the patients gave informed consent to participate in the study. (2) Exclusion criteria: ① the expected operation time was less than 1 hour; ② patients with central high fever, dysthermoregulatory diseases, infectious fever, and temperature >38°C 3 days before surgery; ③ confirmed infection before surgery; and ④ suspected infection without definite diagnosis before surgery.

### 2.3. Materials

**Figure 1:**
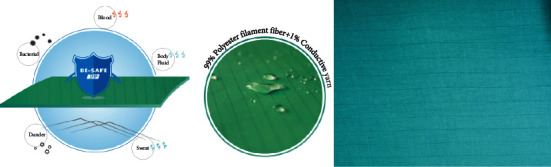
Schematic diagram of the characteristics of long fiber polyester fabric drapes.

**Figure 2:**
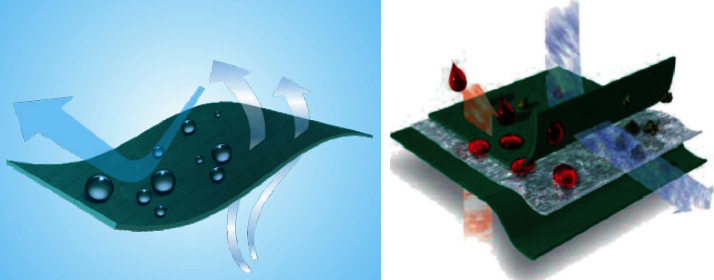
Schematic diagram of microporous technology.

**Figure 3:**
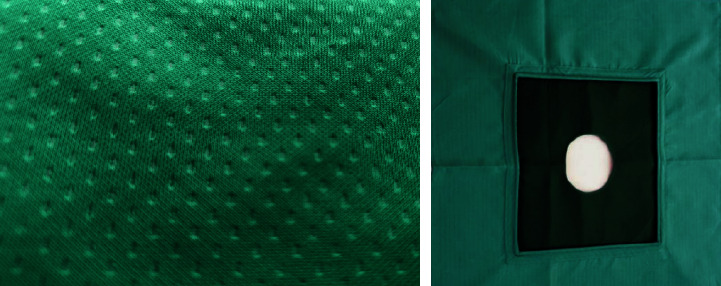
Water absorbing and wear-resisting functional materials used in the critical area of the reusable surgical towels.

The surgical drapes of the study group were long-fiber polyester fiber cloth, which is a plain and rectangular large towel woven material specially designed for medical institutions. It has passed the strict testing of European EN13795 and American AAMI PB-70 specifications, which also meet the pharmaceutical industry standard YY/T 0506.8-2019 of the People's Republic of China. The long fiber polyester fiber has a warp density of 150–170 roots/share and a weft density of 80–100 roots/share, it also has the texture of traditional cotton cloth, with hydrophobicity, without flocculation and dust, excellent waterproof effect, and high strength of resistance tear and burst, material skid resistance designed, and lightweight, not easy to dye, low cost of washing and sterilization, and good air permeability, with conductive fiber inserted every 0.3–0.8 cm of the width of the drapes to make them have an antistatic effect. After 100 reuses, hydrostatic pressure resistance ≥100 cm H_2_O can effectively block the penetration of blood, bacteria, and even viruses and cause the hot air or water vapor produced by the skin to be discharged from the inside, maintaining excellent characteristics of physiological comfort. It can be sterilized by high-temperature and high-pressure steam. The critical zone approximately 50 cm from the incision was covered with three layers of the long-fiber polyester fiber cloth, and the noncritical zone was covered with a single layer of this new surgical drapes. These three layers of composite material provide a high level of waterproofing and bacterial protection effects. They are also reusable drapes with a protection level of 4 (according to the manufacturer information), in which the outer layer is woven of microdenier yarns (long fiber polyester fiber), namely, the water-absorbing layer, the rate of water absorption performance is more than 75% of the drop of water, and with softness and draping quality. In addition, it can prevent liquid backflow around the incision during surgery, preventing incision site infection. The middle layer contains a porous membrane with a waterproof layer (polyurethane, polytetrafluoroethylene, and microporous film), which possesses antivirus, antialcohol, and other chemical solvent penetration. The inner layer is a knit fabric (long fiber polyester fiber) that is breathable, has no flocculation, and is antistatic. The constituent yarns of both the outer and inner layers are composed of 99% PES/1% carbon fibers (Figures 1–3).

**Figure 4:**
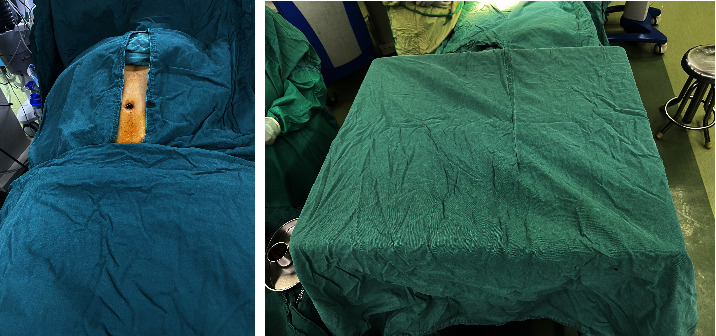
Diagram of cotton surgical drapes.

The surgical drapes of the control group were conventional cotton surgical drapes. It is a cotton rectangular towel, which can be used about 20 times under normal circumstances. The pressure steam sterilization method can be used for disinfection and sterilization. The cloth had to be kept dry to be resistant to bacterial penetration and was not waterproof, with a water absorption capacity of 15% (Figure 4).

### 2.4. Surgical Drapes Laying Methods

**Figure 5:**
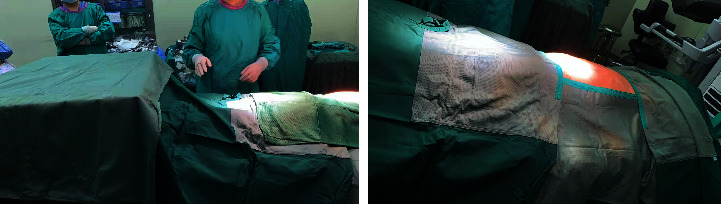
Diagram of laying surgical drapes.

After surgical preparation, povidone iodine disinfectant with an effective iodine concentration of 5000 mg/L was used for skin disinfection at the surgical site and prophylactic use of a reasonable dose of antibiotics 30 min before surgery. The study group received long-fiber polyester fiber surgical drapes. Spreading the sterile surgical drapes, and pasting on the contact skin surface using the adhesive (Figure 5). The control group was covered with a conventional cotton surgical cloth, which had three layers of cotton cloth within 20 cm around the incision, and was spread under sterile conditions after sterilization. The laying process of cotton cloth surgical drapes was consistent with the basic method of long polyester fiber surgical drapes; because of its heavy weight and good drapability, it was not pasted on the skin of the surgical patient. Instead, the surgical incision hole towels were clamped and fixed with towel laying pliers or fixed by pasting sterile film. Before the two types of surgical drapes were spread, unified training for surgeons and nurses to spread the drapes during the operation did not cut the surgical drapes due to the extension of the incision or tighten the size of the drape hole due to the shrunken incision. Operating rooms were cleaned with laminar flow purification.

### 2.5. Measurements

#### 2.5.1. Primary Outcome Measures

Liquid permeability rate of surgical drapes (whether the surgical drapes were wet with liquid, seepage rate = number of cases of liquid infiltration/total number of cases × 100%).

#### 2.5.2. Secondary Outcome Measures

Incision infection rate within 1 week after surgery (the diagnostic criteria for surgical incision infection were judged according to the diagnostic criteria for surgical incision infection issued by the National Health Commission of China [25], which detailed the following: redness, induration, or tenderness within 2 cm of the incision site, positive exudate culture, elevated body temperature, elevated white blood cells and neutrophils), cost effect of surgical drapes (calculate the average per use cost, in U.S. dollars), water absorption performance (water dripping on the drapes until dripping/cm^2^), antislipping rate of the surgical drapes (surgical instrument drop rate), accidental injury of patients (such as electric scalding) during the surgery, average weight of the drape package (kg), feeling of nursing staff (comfort level of use: very comfortable, general, not comfortable, self evaluation by nursing staff), and average time consumption of surgical drape spreading (min). In addition, the use of antibiotics, duration of operation (min), intraoperative irrigation (ml), length of stay (day), readmission rate within 1 month after operation (%), and hospitalization cost ($) were compared between the two groups.

### 2.6. Data Analysis

SPSS 26.0 was used for statistical analysis, a *P*-value of <0.05 was considered statistically significant. The mean ± standard deviation ($\bar{X}$ ± SD), median (*M*), quartile range (QR), frequency, or rate was used for the statistical description. Student's *t*-test, Wilcoxon test, ANOVA, LSD, Kruskal‒Wallis *H* test, chi-square test (*χ*^2^), rank sum test, or Fisher's test was used for statistical inference.

### 2.7. Ethical Approval and Informed Consent

Ethics approval was obtained from the Biomedical Research Ethics Committee of West China Hospital of Sichuan University on 7 July 2022 (No. 2022–874), and written consent was obtained from every participant. The study was registered at the Chinese Clinical Trail Registry (ChiCTR2200064240) on October 1, 2022. The full study protocol can be accessed at https://www.chictr.org.cn/edit.aspx?pid=169802&htm=4.

## 3. Results

### 3.1. Patient Characteristics

**Figure 6:**
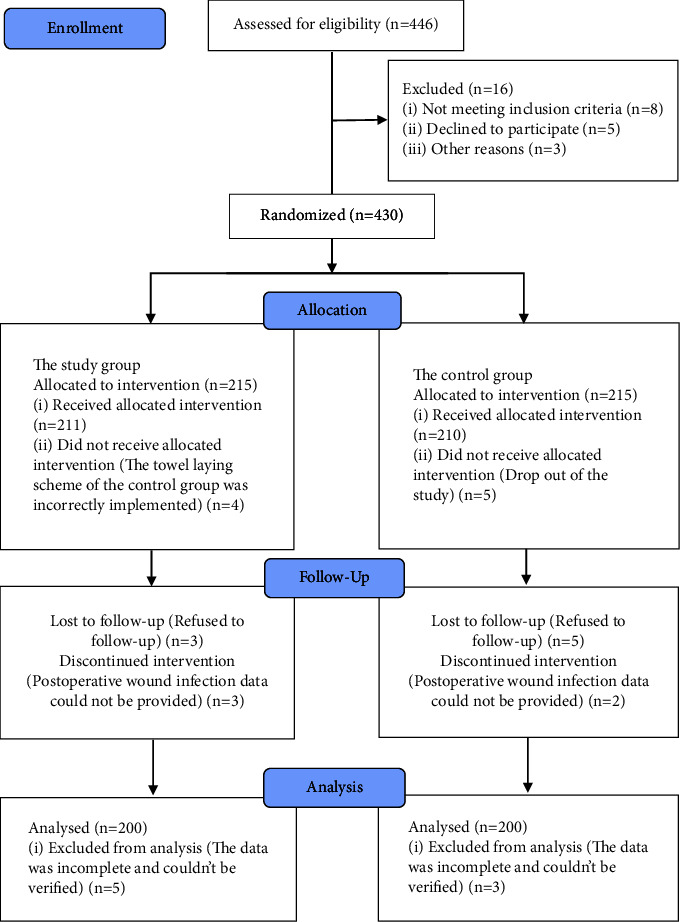
CONSORT 2010 flow diagram.

A total of 400 patients were finally included and analyzed in this study, 200 in the study group and 200 in the control group (Figure 6). There were 103 males and 97 females in the study group, with an average age of 48.2 ± 5.7 years. There were 107 males and 93 females in the control group, with an average age of 47.2 ± 6.2 years. There were no statistically significant differences in the clinical data of the two groups, such as body mass index (BMI), diagnosis, number of people in the operating room, number of medical staff on the operating table, operation time, and amount of intraoperative flushing fluid (*P* > 0.05), as shown in Table 1.

### 3.2. Comparison of the Application Effect of Long-Fiber Polyester Surgical Drapes and Conventional Cotton Surgical Drapes in the Sterile Operation Interface of Surgical Critical Area

Compared with cotton surgical drapes, the long-fiber polyester surgical drapes had some advantages, the difference was statistically significant (*P* < 0.05). Of which the rate of SSI within one week after surgery, the number and rate of wet surgical drapes, the drop number of surgical instruments from surgical drapes surface, number of skin scald in surgical patients, the average time consumption of surgical drape spreading (min), readmission rate within one month after surgery were all reduced, and the average weight of the drape package (kg) was lighter, while the water absorption performance and the comfort level of use evaluated by the nursing staff were superior. Other comparative items, such as number of skin scald in surgical patients (*n*), length of stay (day), and hospitalization cost ($), were not statistically significant (*P* > 0.05) (Table 2).

### 3.3. Comparison of the Use and Disinfection Cost of Different Surgical Drapes

As shown in Table 3, although the initial cost of long fiber polyester surgical drapes was higher than cotton cloth and disposable nonwoven fabric cloth, the average total cost per use was the lowest, thus providing a cost-effective advantage.

## 4. Discussion

### 4.1. Long Fiber Polyester Surgical Drapes is Safer for Patients and Surgical Personnel due to the Better Resistance to Liquid Penetration

As illustrated in Table 2, compared with cotton surgical cloth, the long fiber polyester surgical drapes had the advantages (*P* < 0.05), which is safer for patients and surgical personnel due to the better resistance to liquid penetration. There were 5 cases of SSI that occurred within one week after surgery in the study group, while it was 16 cases in the control group, rate of SSI within one week after surgery was lower in the long fiber polyester surgical drapes group (2.5% vs. 8.0%, *χ*^2^ = 6.081, *P*=0.014). Because of its special structure and material properties, the long fiber polyester surgical drapes was made by the high-density weaving method with warp and weft densities of approximately 150–170 and 100 roots/share, respectively [26], which has good water resistance, excellent hydrostatic pressure resistance (the average hydrostatic test index of the long-fiber polyester drapes was 86.52 ± 0.62 N/m^2^, which was higher than that of the cotton fabric group (11.53 ± 0.53 N/m^2^) (*P* < 0.001) [24], and good antipermeability (approximately 10 times that of cotton surgical towels) [19]. Consequently, it can effectively block bacteria and dust particles from the environment into the incision of surgical patients. In addition, because the long fiber polyester surgical drapes have strong toughness, the surface has a certain antisplashing performance, the moist gas and water in the air will not condense and remain on the fiber surface of the material, and there is no water in the gap between fiber bundles, so it is difficult to form bacterial pathways inside and outside the material, resulting in bacteria inhibition [27]. These functions and characteristics enable the long-fiber polyester drapes to form a good microbial barrier.

Prior studies showed that skin bacterial contamination at the surgical site is correlated with incision infection [25, 28]. More than 20% of skin bacteria exist in skin hair follicles and sweat glands, and local skin disinfectants cannot completely remove these bacteria. Surgical drapes are used to prevent the resident bacteria in the skin hair follicles and sweat glands from entering the surgical incision and minimize the contamination by microorganisms [29]. One of the basic principles of the American Association of Operating Room Nurses (AORNs) guidelines for aseptic surgical techniques is to cover the patient's surgical site and necessary medical instruments during surgery with aseptic surgical towels [30]. The Chinese State Food and Drug Administration implemented special requirements for surgical sheets in August 2020, and industry standards for surgical drapes, gowns, and clean clothes for patients, medical staff, and instruments have been developed [31]. They have all emphasized that good surgical drapes can effectively prevent microbial contamination of the surgical site. Traditional cotton surgical drapes have the advantages of good adhesion, drapability, and reusability [32]. However, there are many hydroxyl groups (OH) in the molecular structure of the cotton fiber, which have polarity and easily absorb water molecules and bacterial liquid. Meanwhile, its macromolecular structure is easily hydrolyzed and releases more nutrients to promote the mass growth and reproduction of microorganisms [33]. In contrast to cotton surgical drapes, a study reported [27] that long-fiber polyester surgical drapes have reached the fourth-grade protection standard and have the characteristics of excellent hydrophobicity, good air permeability, and they have a good protective effect against bacterial contamination in surgical incisions compared with cotton drapes [20], which is conducive to the prevention and control of SSI.

In addition, due to the good barrier liquid penetration function of the long fiber polyester surgical drapes, so as to effectively reduce the rate of wet surgical drapes (835 ± 15.8 vs. 225 ± 21.0, *t* = 328.261, *P* < 0.001), thus reducing the penetration of bacteria, viruses, and other microorganisms in the wet environment and because of the liquid penetration bring away the patient heat to reduce the patient's body temperature, which can effectively reduce the rate of SSI within one week after surgery (2.5% VS 8.0%, *χ*^2^ = 6.081, *P*=0.014) and readmission rate within one month after operation (1.0% VS 6.0%, *χ*^2^ = 7.402, *P*=0.007). The flushing fluid can carry the patient's blood and body fluids, which soaked patients, surgery clothes, and bed units, thereby increasing the risk of cross-infection. In our study, the penetration rate of long-fiber polyester surgical drapes was significantly lower than that of cotton surgical drapes (2.5% VS 37.5%, *χ*^2^ = 76.562, *P* < 0.001). Yu et al. [34] study showed that the use of long-fiber polyester surgical drapes was superior to cotton cloth in reducing the incidence of hypothermia and the postoperative infection rate of patients. A study illustrated that compared with traditional cotton surgical drapes, long-fiber polyester surgical drapes can significantly reduce the rate of dressing wetting in emergency cesarean and maternal heat loss [21]. Liquid blocking protection helps prevent hypothermia, further preventing the occurrence of postoperative incision infection.

Surgical drapes are applied throughout the surgical process to ensure the safety of patients and medical staff and avoid cross-infection [35, 36]. The quality of surgical drapes will directly or indirectly affect the risk of infection [37]. China's GB/T19633 final sterilized medical device packaging [38, 39] and YY/T0698 final sterilized medical device packaging material [40] have specific requirements for the performance of medical packaging materials: (1) the material itself should not have floc; (2) materials in dry and wet conditions should have the ability to block microbial penetration; (3) the material should have certain air permeability; (4) the pH value of the material after treatment is neutral, and there should be no chemical residue and fluorescence. Long fiber polyester cloth has the characteristics of waterproof, breathable, high strength, and no floc production, which meet the above-mentioned specifications and requirements [27]. The results of our study confirmed that long-fiber polyester surgical drapes have a better barrier effect on bacteria in the same operating environment and mode. In addition, triple-layer surgical drapes were used in the critical zones to meet various desired objectives: the outer layer was designed to resist abrasion and puncture, the middle layer provided exceptional barrier resistance to fluid penetration, and the soft bottom layer added comfort in addition to another layer of protection [41]. A study has shown that storage of long-fiber polyester surgical drapes at an appropriate temperature has a longer validity period than cotton surgical drapes [42]. Chen et al. [43] study showed that cotton surgical drapes were prone to droop in the process of laying surgical drapes but increased the number of dust particles in the air, while long fiber polyester surgical drapes had the characteristics of being smooth and soft, having a high filtration rate, effectively controlling SSI, which was similar to the results of our study. This proved that long-fiber polyester surgical cloths are more suitable for surgery.

### 4.2. Using Long Polyester Fiber Surgical Drapes Can Improve the Efficiency and Cost Effect of Surgical Towel Placement

The long fiber polyester surgical drapes were used, the drop number of surgical instruments from surgical drapes surface (7.5% VS 17.0%, *χ*^2^ = 8.396, *P*=0.004), number of skin scald in surgical patients (0 VS 1, Fisher *P*=1.0), and the average time consumption of surgical drape spreading (min) (0 VS 1, Fisher *P*=1.0) were all reduced, and the average weight of the drape package (kg) was lighter (3.5 ± 1.6 VS 4.6 ± 1.3, *t* = −7.546, *P* < 0.001), while the water absorption performance and the comfort level of use evaluated by the nursing staff (67.5% VS 52.0%, *χ*^2^ = 9.99, *P*=0.002) were superior (Tables 2 and 3). Whether disposable or reusable of surgical drapes, is considered a protective barrier to prevent infection from spreading. The selection of disposable or reusable drapes should be based on the protection performance, environmental impact, and economics [13]. Long fiber polyester drapes can be used repeatedly more than 100 times [44], and the environmental benefits of using reusable drapes due to their reprocessing ability enable minimization of the quantity of clinical waste, which can achieve substantial cost savings both in terms of incineration and is essential to maintain a stock of single-use materials [45]. Although the initial cost of long fiber polyester surgical drapes was higher than cotton cloth and disposable nonwoven fabric cloth, the average total cost per use was the lowest (0.83 VS 0.96–1.09, dollars), which can be reused repeatedly to reduce the cost of hospital use and posttreatment, thus providing a cost-effective advantage. In addition, the long fiber polyester surgical drapes were used can reduce the number of towels spreading layers, improve the fixation method, simplify the drape spreading steps on the premise of following the principle of sterility, save the time of drape spreading, save human and material resources to a certain extent, and improve work efficiency [46, 47].

Meanwhile, a conductive carbon fiber is inserted into the long-fiber polyester drapes every approximately 0.5 cm so that it has the high antistatic ability. Besides, the elimination of static electricity on the surface of the material can prevent static electricity from absorbing suspended dust and foreign matter in the air and ensure the clean surface of the surgical drapes [48]. In our study, there was no skin scald of surgical patients in the long fiber polyester surgical drapes group, but in the cotton cloth group, because the electric knife was placed on the surgical drapes, the surgeon mistakenly activated the electric knife, leading to the skin scald of one surgical patient. The reason for the analysis is mainly the doctor's careless wrong operation, followed by the long fiber polyester cloth due to the addition of conductive fiber in the fiber layer of the cloth, has electrical conductivity and antistatic effect, while cotton cloth does not have these functions, especially under the wet condition is easier to conduct electricity, the cotton cloth surgical drapes is relatively less safe for patients. On the other hand, the surface of long fiber polyester surgical drapes has a special design of coarse grain small squares (Figure 3), resulting in uneven material surface, friction coefficient increases, so it has a certain degree of nonslip, can effectively prevent slipping of the surgical instruments on the drape surface, thus the drop rate of surgical instruments from surgical drapes surface was decreased in the study group (7.5% VS 17.0%, *χ*^2^ = 8.396, *P*=0.004). They all proved that the long-fiber polyester drapes was more safer to be used.

Under the condition of the same protective effect, the number of the layer was reduced, so the weight of long fiber polyester surgical drapes was lighter than that of cotton cloth surgical drapes, which was convenient for surgical personnel to lay towel and reduced the work burden of the surgical personnel. Besides, surgical care staff prefers to use long polyester fiber surgical drapes because of their antifluid penetration and air permeability, the surgical nursing staff self-rated comfort of use was very comfortable at 67.5% VS 52.0% (*χ*^2^ = 9.99, *P*=0.002). To sum up, it shows that long fiber polyester fiber surgical drapes have advantages: safety, convenience, economy, and comfort.

### 4.3. Limitations

There are some limitations in our study: (1) this study was conducted in a tertiary hospital and a surgical department, thus the extrapolation of its conclusions is limited. The reference of this study's results should be based on the cultural and economic conditions of each hospital. (2) Because bacterial sampling and analysis at surgical incisions were not performed and bacterial species at surgical incisions were not analyzed, only the incidence of SSI during the first week after surgery was analyzed in this study, failing to conduct an in-depth exploration of the research results. In the future, it is necessary to conduct a bacterial community analysis of samples around the incision to further explore the bacteria-blocking effect of different surgical towels and the protective effect on patients and surgical staff.

### 4.4. Implications for Nursing Management

Laundry and sterilization processes lead to the destruction of the single-layer drape structure and enhance its pore size, followed by barrier index reduction. While the long fiber polyester cloth was superior to conventional cotton cloth in water absorption, preventing device slip and preventing patients from burning, and which has a stronger antipenetration ability of microorganisms and forms an effective protective barrier to ensure the sterility of surgical drapes, and its cost effect is superior. Operating room nursing managers can introduce long fiber polyester drapes into the selection of medical textiles to construct aseptic surgical barriers and prevent SSI, and it is important to surgical staff and patients to reduce the cross-infection, providing a better surgical towel scheme, rationally selecting medical wrapping cloths, and reducing surgery-related medical costs.

## 5. Conclusions

Compared with cotton fabric, long-fiber polyester fabric has a stronger antipenetration ability of microorganisms and forms an effective protective barrier to ensure the sterility of surgical drapes. It also has strong hygroscopicity, and its special design can prevent the occurrence of adverse events such as sliding of surface instruments and burns of patients, can effectively ensure the safety of patients and medical staff in the process of surgery. In addition, its cost effect is superior. Therefore, long-fiber polyester surgical drapes are more suitable for clinical use and can be used repeatedly with good application prospects.

## Figures and Tables

**Table 1:** Comparison of clinical data between the two groups (*N* = 400).

Comparative items	The study group (*n* = 200)	The control group (*n* = 200)	Statistical value	*P* value
Gender (*n*)	Male (107), female (93)	Male (102), female (98)	*χ* ^2^ = 0.251	0.617
Average age (years) ($\bar{X}$±SD)	65±10.5	63±11.2	*t* = 1.842	0.066
BMI (kg/m^2^) ($\bar{X}$±SD)	23.42±1.28	23.51±1.15	*t* = −0.74	0.460
Diagnosis (*n*)	200	200	*χ* ^2^ = 0.16	0.689
Kidney cancer	40	38		
Bladder cancer	57	55		
Urethral stones	43	48		
Prostate cancer	35	30		
Ureteral stricture	12	14		
Uterine prolapse	13	15		
ASA score (*n*)	Grade II (34), grade III (166)	Grade II (27), grade III (173)	*χ* ^2^ = 0.948	0.33
The number of people in the operating room ($\bar{X}$±SD)	9±1.2	9±1.5	*t* = 0.00	1.000
The number of medical staff on the operating table ($\bar{X}$±SD)	5±1.5	5±2.3	*t* = 0.00	1.000
Duration of operation (min) ($\bar{X}$±SD)	135±45	142±53	*t* = −1.424	0.155
Amount of intraoperative flushing fluid (ml) ($\bar{X}$±SD)	1455±185	1462±152	*t* = −0.413	0.680
Intraoperative bleeding (ml) ($\bar{X}$±SD)	126±25	122±28	*t* = 1.507	0.133
Time of preoperative antibiotic use (min) ($\bar{X}$±SD)	16±6.0	17±5.0	*t* = −1.811	0.071

**Table 2:** Comparison of two groups of surgical drapes in the prevention of adverse events in patients undergoing surgery (*N* = 400).

Comparative items	The study group (*n* = 200)	The control group (*n* = 200)	Statistical value	*P* value
Rate of surgical site infections within one week after surgery (*n*, %)	5 (2.5%)	16 (8.0%)	*χ* ^2^ = 6.081	0.014
The number and rate of wet surgical drapes (*n*, %)	5 (2.5%)	75 (37.5%)	*χ* ^2^ = 76.562	<0.001
Water absorption performance (g/m^2^) ($\bar{X}$ ± SD)	835 ± 15.8	225 ± 21.0	*t* = 328.261	<0.001
The drop number of surgical instruments from surgical drapes surface (*n*, %)	15 (7.5%)	34 (17.0%)	*χ* ^2^ = 8.396	0.004
Number of skin scald in surgical patients (*n*)	0	1		Fisher *P*=1.0
Average weight of the drape package (kg) ($\bar{X}$ ± SD)	3.5 ± 1.6	4.6 ± 1.3	*t* = −7.546	<0.001
The feeling of nursing staff (*n*, %)	Comfort level of use: very comfortable (135, 67.5%) general (43, 21.5%) not comfortable (22, 11.0%)	Comfort level of use: very comfortable (104, 52.0%) general (65, 32.5%) not comfortable (31, 15.5%)	*χ* ^2^ = 9.99	0.002
The average time consumption of surgical drape spreading (min)	9.3 ± 1.5	9.8 ± 1.8	*t* = −3.018	0.003
Length of stay (day) ($\bar{X}$ ± SD)	6.5 ± 1.3	6.7 ± 1.2	*t* = −1.599	0.111
Readmission rate within 1 month after surgery (*n*, %)	2 (1.0%)	12 (6.0%)	*χ* ^2^ = 7.402	0.007
Hospitalization cost ($)	1105.5 ± 112.5	1112.2 ± 121.3	*t* = −0.573	0.567

**Table 3:** Comparison of the use and disinfection cost of different surgical drapes.

Texture of material	Initial cost (dollar/pack)	Useable times	Laundry cost	Packing and sterilization (dollar/time)	Total cost per use (dollar/time)
Cotton	11.86–14.83 ($)	20 times	0.26 dollar/piece	0.08 ($)	0.96–1.09 ($)
Long fiber polyester fiber	59.32 ($)	100 times	0.16 dollar/piece	0.08 ($)	0.83 ($)
Disposable non-woven fabric	2.97–11.86 ($)	1 time	Waste disposal 1.34 dollar/kg (0.42 kg/piece)		3.53–12.43 ($)

## Data Availability

The data used to support the study are available from the corresponding author upon request. The data are not publicly available because of privacy or ethical restrictions.
